# The Considerable Water Evaporation Induced by Human Perspiration and Respiration in Megacities: Quantifying Method and Case Study in Beijing

**DOI:** 10.3390/ijerph19148638

**Published:** 2022-07-15

**Authors:** Chuang Liu, Jiahong Liu, Weiwei Shao, Jiahui Lu, Han Gao

**Affiliations:** 1 State Key Laboratory of Simulation and Regulation of Water Cycle in River Basin, China Institute of Water Resources and Hydropower Research, Beijing 100038, China; liuchuang1619@163.com (C.L.); shaoww@iwhr.com (W.S.); alujiahui@163.com (J.L.); 2 Engineering and Technology Research Center for Water Resources and Hydroecology of the Ministry of Water Resources, Beijing 100038, China; 3 Key Laboratory of River Basin Digital Twinning of Ministry of Water Resources, Beijing 100038, China; 4 College of New Energy and Environment, Jilin University, Changchun 130021, China; gaohanhan18@163.com

**Keywords:** human body evaporation, water resources, urban hydrological cycle, evapotranspiration, Beijing

## Abstract

The water cycle in urban areas is called the natural-social dualistic water cycle, and it is driven not only by natural forces, but also by human activities. As the drivers of the social water cycle, human perspire continuously, and this is often overlooked as a contributing factor to the water cycle. This paper proposes a method for quantifying the water evaporation induced by human perspiration and respiration in megacities. A calculation based on the sweating prediction model was applied to the city of Beijing to evaluate the evaporation from the human body. The results show that the greatest volume of evaporation produced by human occurs in summer, and the least in spring. The total evaporation produced by human was converted to the evaporation on unit area of the city and reached 5075.2 m^3^/km^2^ in the six core districts of Beijing. According to the calculation, the total volume was considerable and reached 14.0 million m^3^ in 2020, which was equivalent to the annual evapotranspiration from an area of 104.9 km^2^ of Acer truncatum forest (15 cm diameter at breast height, afforestation density 800 plants/hm^2^), and even twice the annual total water use in Tartu, Estonia. The results of the study provide a reference for dualistic water cycle research and water cycle flux calculation in urban areas.

## 1. Introduction

Cities are a result of the development of human society and are the centers of the majority of a country’s population and its wealth. With the rapid development of the economy, the process of urbanization is accelerating [1]. According to the forecast of the United Nations Population Division, the urbanization rate of every developing country or region in the world will exceed 50% in 2030, and two-thirds of the population will live in cities by 2050 [2]. With the rapid urbanization process, human beings continue to congregate in urban areas, and urban transformation activities are becoming more frequent [3,4], altering the original water cycle system from a single cycle dominated by nature to a cycle dominated by both nature and human society, which is called a natural-social dualistic water cycle system [5]. As an essential part of the urban water cycle process, urban regional evapotranspiration [6,7] is an important source of urban water vapor, and it has a significant impact on urban microclimate changes and water cycle flux calculations [8], including outdoor natural water evaporation and indoor water dissipation from humans [9,10,11]. Evaporation means the phase transition of water from liquid to gas, which is driven by liquid water, the available energy to meet the latent heat requirements of the phase transition, and the vapor pressure gradient between the evaporation surface and the atmosphere [12]. Evaporation plays a decisive role in various water resource management activities, and the quantification of regional evaporation is a basic requirement for water balance calculations [13]. The quantification of evaporation can be based on methods such as noting the liquid water that is removed by the water balance, noting the energy allocated to latent heat using the energy balance, or by the assessment of water vapor flux [12]. Pereira et al. [14] dissected the concept of evaporation, reviewed from Penman to Penman–Monteith methods, and introduced the standard concepts of potential evaporation (EP) and equilibrium evaporation (Ee). Dash et al. [13] used SWAT and VIC models to predict evaporation at the watershed scale and compared the prediction accuracy and applicable conditions of each model. Based on the improved Penman–Monteith (PM) method, Chen et al. [15] established a hybrid deep learning (DL) model that can describe the physical process to simulate and predict evaporation, and the prediction results were better than the six classical machine learning models. Most of the natural evapotranspiration in urban areas is derived from water surfaces and green areas, such as trees, grass, and other vegetation coverage [16]. The calculation of the area of evapotranspiration is extensively used in soil–plant–atmosphere system models [17,18] and remote sensing inversion [19], while the research of plant transpiration water consumption mainly adopts experimental and analytical methods [20,21]. Indoor social water consumption refers to the water dissipation generated through human production and day-to-day activities indoors, which is characterized by the transformation of water from liquid to gas, with only minor research conducted to date on this aspect. Some studies have focused on the calculation method of water consumption inside buildings and developed a building water dissipation model considering the indoor population and total floor area [22]. Along with the in-depth study, an urban water dissipation calculation model [16] was proposed and applied to the cities of Beijing [8] and Xiamen [9], and the characteristics of urban water dissipation changes that consider the water dissipation inside buildings were analyzed. However, when humans carry out water dissipation activities indoors, they also perspire at the same time. With the acceleration of urbanization and the increase in the urban population, the evaporation from humans cannot be ignored.

Water evaporates from human bodies mainly through the skin and breathing, and this is generally divided into “insensible perspiration” and “sensible perspiration” [23]. “Insensible perspiration” is the evaporation from the human body caused by the diffusion of water on the skin’s surface and the exchange of water vapor in the respiratory system. It occurs continuously and is unnoticeable [24]. When the ambient temperature rises or the activity intensity increases, the heat emitted by the human body through radiation and convection is unable to eliminate the heat generated by metabolism, so the sweat glands begin to secrete sweat to maintain body heat balance; this visible sweating phenomenon is called sensible perspiration [25]. To study the mechanism of human sweating, many researchers have carried out experimental tests on the amount of human sweat and local sweating. Test methods that have been commonly used include the weighing method [26], vinyl bag sweat collection method [27], gauze sponge pad method [28], ventilated sweat sac method [29], sweat patch method [30], filter paper method [31], etc. Based on the research and analysis of the test results, some researchers [32] believe that the quantity of human sweat is related to the evaporation required to maintain heat balance at any given core temperature and to the maximal evaporative capacity of the environment from the body, and they constructed a sweat prediction equation called the original Shapiro prediction equation (OSE). Gonzalez et al. [26] improved on the theoretical basis of Shapiro, extended the OSE to low-temperature environmental conditions, and established a piecewise (PW) sweat prediction equation (which was based on fuzzy PW regression analysis) with a wider range of applications. Based on the above research, it can be found that the research on evaporation has mainly focused on research fields such as vegetation and soil in nature, few studies have paid attention to the amount of evaporation produced by the human body, and while there have been in-depth studies on the entire and localized sweating of the human body, there are few studies on human body evaporation on an urban scale from the perspective of artificial water dissipation.

Based on mathematical models, this study used the city of Beijing as the study area and calculated the evaporation generated by the human population in 2020. According to the population data of each district, the spatial distribution of human body evaporation was analyzed. To quantify the evaporation generated by the human body in 2020 more intuitively, it was compared with the annual forest evaporation and the total annual water consumption in some cities in Europe. The research results provide a reference for the study of the urban dualistic water cycle.

## 2. Study Area and Methods

### 2.1. Study Area

**Figure 1:**
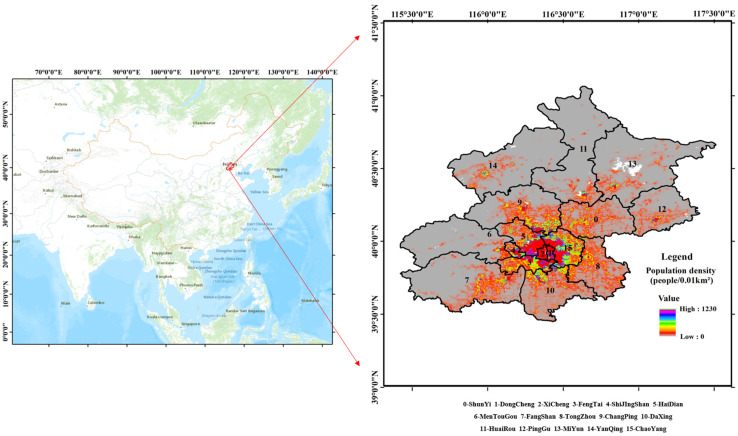
Regional division and population density distribution of Beijing in 2020.

Beijing is the capital, the political and cultural center of China. It is located at the junction of the Taihang and Yanshan Mountains in the North China Plain. The latitude and longitude of Beijing are 115.7–117.4° E and 39.4–41.6° N, respectively. Beijing falls within the semi-humid continental monsoon climate of the northern temperate zone, with four distinct seasons. Its annual average temperature is 10–13 °C, and the annual average rainfall is 589 mm. The rainfall is unevenly distributed throughout the year, mainly concentrated in July and August. The rainfall in these two months accounts for 60% of that of the whole year, and the rainfall from May to August accounts for 82% of that of the whole year, with low precipitation in spring and winter, with frequent droughts. The average annual evaporation from 2003 to 2012 was 517 mm [33], and evaporation is greatest in summer and least in winter. According to the Beijing Regional Statistical Yearbook 2020, at the end of 2020, Beijing comprises 16 districts, with a total area of 16,410.54 km^2^, and the permanent population was 21.89 million, making it one of the most populous cities in China. The population was mainly concentrated in the six core districts of the city. The location and population density distribution of each district is shown in Figure 1. The population density data were obtained from the world population database (https://www.worldpop.org/, accessed on 15 January 2022), with a resolution of 100 m × 100 m.

### 2.2. Methods

Regarding the amount of evaporation generated by the human body during daily activities, in 2009, Gonzalez et al. established the PW sweat prediction equation [26] as follows:

(1) $$m_{\mathrm{sw}}=147+1.527\times E_{\mathrm{req}}-0.87\times E_{\max}$$

where m_sw_ is the sweating rate, g·m^−2^·h^−1^; E_req_ is the evaporation required to maintain heat balance at a given core temperature, and E_max_ is the maximal evaporative capacity of the environment, W/m^2^. The amount of sweating for one human can be calculated by m_sw_ × body surface area × time. Furthermore, the amount of human sweating in each district can be calculated by multiplying the population of each district.

The equation of the evaporation required to maintain heat balance at any given core temperature is shown as:

(2) $$E_{\mathrm{req}}=M-W-C-R-Q_{\mathrm{res}}$$

where M is the metabolic rate, which depends on the amount of human activity, W/m^2^; W is the rate of accomplished mechanical work, W/m^2^; C is the rate of convective heat loss, W/m^2^; R is the rate of radiant heat loss (or gain) from the surrounding surfaces, W/m^2^; and Q_res_ is the human breathing heat dissipation, W/m^2^.

The equation of the metabolic rate is shown as:

(3) $$M=\frac{21(0.23\mathrm{RQ}+0.77)V_{O2}}{A_{D}}$$

where RQ is the molar ratio of exhaled carbon dioxide and inhaled oxygen per unit time, dimensionless; V_O2_ is the volume of oxygen consumed per unit time under the conditions of 0 °C and 101.325 kPa, mL/s; and A_D_ is the surface area of naked human skin, m^2^.

The equation of the rate of accomplished mechanical work is shown as:

(4) W=η×M

where η is the mechanical efficiency, which is generally 5–10% under different activity intensities. For most activities, the mechanical efficiency of the human body is almost 0.

The equation of the rate of convective heat loss is shown as:

(5) $$C=f_{\mathrm{cl}}h_{c}\left(t_{\mathrm{cl}}-t_{a}\right)$$

where f_cl_ is the clothing area factor; h_c_ is the convective heat transfer coefficient, W/(m^2^·K); t_cl_ is the clothing surface temperature, K; and t_a_ is the air temperature around the human body, K.

The equation of the rate of radiant heat loss from the surrounding surfaces is shown as:

(6) $$R=f_{r}h_{r}\left(t_{\mathrm{cl}}-t_{r}\right)$$

where f_r_ is the effective radiation area factor; h_r_ is the linear radiation transfer coefficient, W/(m^2^·K); and t_r_ is the ambient average radiant temperature, K.

The equation of the human breathing heat dissipation is shown as:

(7) $$Q_{\mathrm{res}}=0.0173M\left(5.867-P_{a}\right)+0.0014M\left(34-t_{a}\right)$$

The equation of the maximal evaporative capacity of the environment is shown as:

(8) Emax=(Psk−Pa)Ie,cl+1fclhe

where P_sk_ is the vapor pressure of water on the skin’s surface; P_a_ is the vapor pressure of the water of the ambient air, KPa; I_e,cl_ is the latent heat transfer and thermal resistance of clothing, m^2^·kPa/W; and h_e_ is the convective mass exchange coefficient on the garment surface.

(9) $$P_{\mathrm{sk}}=\exp\left(18.6686-\frac{4030.183}{T_{\mathrm{sk}}+235}\right)$$

where T_sk_ is the mean skin temperature, °C, which can be estimated using Saltin’s equation [34]:

(10) $$T_{\mathrm{sk}}=0.215T_{a}+26.6$$

where T_a_ is the air temperature, °C.

The equation of the latent heat transfer and thermal resistance of clothing is shown as:

(11) $$I_{e,\mathrm{cl}}=\frac{I_{\mathrm{cl}}}{i_{\mathrm{cl}}\mathrm{LR}}$$

where I_cl_ is the thermal resistance of clothing, m^2^·K/W; i_cl_ is the water vapor permeability coefficient of clothing; and LR is the Lewis ratio, °C/kPa. For a typical indoor air environment, the Lewis ratio is 16.5.

The equation of the convective mass exchange coefficient on the garment surface is shown as:

(12) $$h_{e}=\mathrm{LR}\times h_{c}$$

**Figure 2:**
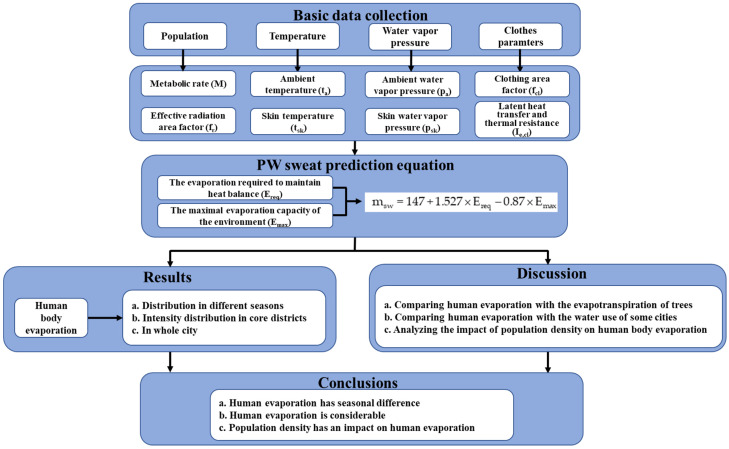
Roadmap of this study.

The following assumptions were made for daily human activities: all activities took place indoors, including 8 h of sleep, 14 h of light work, and 2 h of moderate work. Since sweating is affected by the metabolic rate, temperature, clothing, and other factors during daytime activities, in our calculation, we used the average daily temperature data and considered the difference in the clothes worn during each season. The PW model was used to calculate the amount of sweating during each activity, and the total amount of perspiration in a whole day was calculated. The parameter values used are shown in Table 1, and the technical roadmap is shown in Figure 2.

## 3. Results

### 3.1. Distribution of Human Body Perspiration in Different Seasons

**Figure 3:**
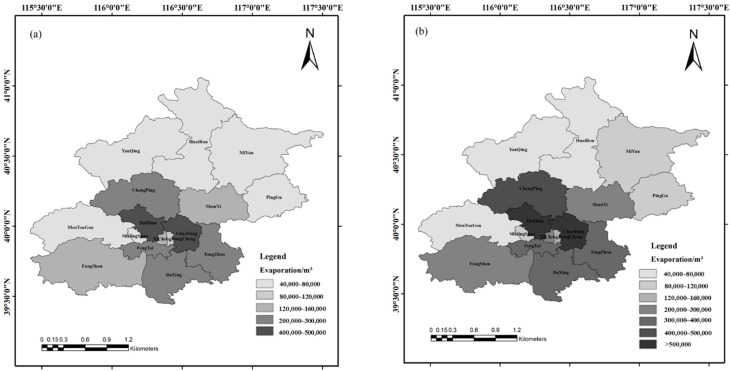

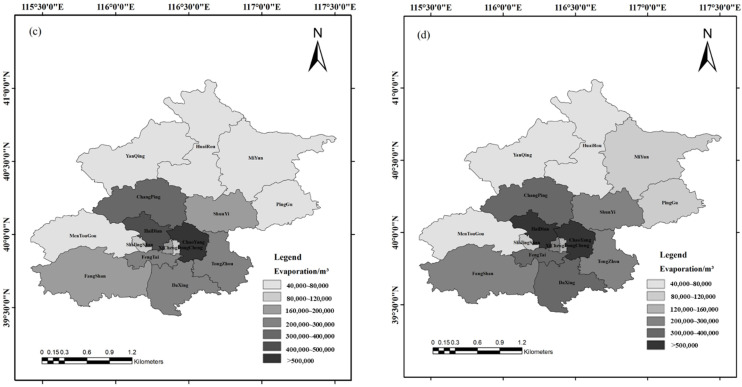
Distribution map of human body perspiration in different seasons of Beijing in 2020. (**a**) Spring; (**b**) summer; (**c**) autumn; (**d**) winter.

Figure 3 shows the human body perspiration in the 16 districts of Beijing over four seasons in 2020. The population of each district differed, so the overall distribution was characterized by the south being more populous than the north, with there also being seasonal differences in each district. The greatest evaporation from humans occurred in summer, followed by winter, autumn, and spring. The temperature in summer is significantly higher than in other seasons, causing the human body to have a higher amount of sweat. Despite the low temperature outside in winter, the central heating in Beijing leads to high indoor temperatures, and people wear more clothes in winter than in other seasons. Moreover, the climate is very dry in winter, with less moisture in the air, making it easier for evaporation to occur, so humans sweat more at the same exercise intensity. The indoor temperature in spring and autumn is obviously lower than that in summer and winter, and humans wear less clothing than in winter, which results in less evaporation from the human body as a whole. It can be seen from Figure 3 that the top two districts where the highest values of human body perspiration occurred were the Chaoyang and Haidian districts. The permanent population of these two districts was much higher than that of other districts, both exceeding 3 million, and perspiration values in each season exceeded 400 thousand m^3^ and more than 500 thousand m^3^ in summer. The district with the third highest value was Changping, with a permanent population of 2.166 million and a human body perspiration total exceeding 200 thousand m^3^ for each season, with a total of more than 400 thousand m^3^ in summer. In addition, the total perspiration rates in the Fengtai, Daxing, and Tongzhou districts exceeded 200 thousand m^3^ for each season, with more than 300 thousand m^3^ in summer. In the Xicheng, Shunyi, and Fangshan districts, it was more than 120 thousand m^3^ for each season, with more than 200 thousand m^3^ in summer. In Dongcheng District, it exceeded 80 thousand m^3^ for every season, with more than 120 thousand m^3^ in summer. Districts with lower figures are in suburbs with a less permanent population, including the Shijingshan, Mentougou, Yanqing, Huairou, Miyun, and Pinggu districts, with the evaporation amount generated in each season less than 120 thousand m^3^.

### 3.2. Distribution of Human Body Perspiration Intensity in Six Core Districts

**Figure 4:**
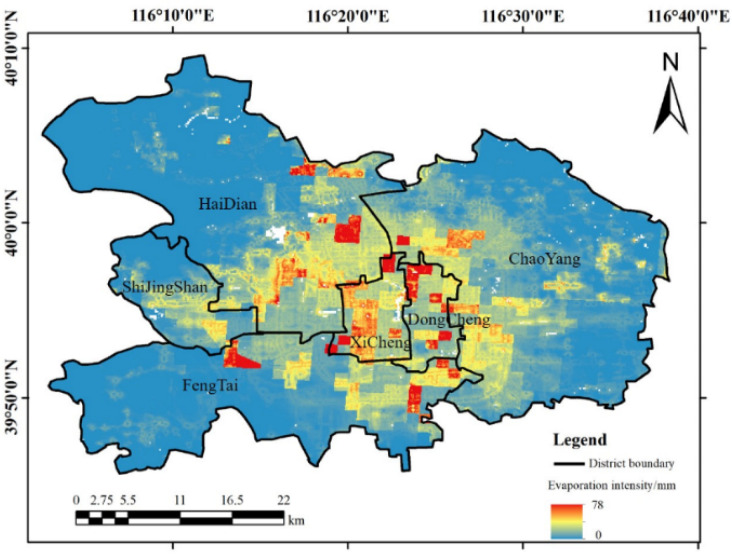
Distribution map of human body perspiration intensity in six core districts of Beijing.

It can be seen from Figure 1 that the population was mainly concentrated in the six core districts of Beijing. Therefore, the annual evaporation produced by the human body in these six districts was calculated and converted into the evaporation intensity per unit area, as shown in Figure 4. We can see that the annual evaporation produced by the human body in the six districts was 0–78 mm/year, and the distribution was uneven. In total, the distribution of human evaporation values in the six core districts showed a gradual decrease from the city center to the surrounding area. The central areas, the Dongcheng and Xicheng districts, maintained high evaporation. The evaporation from Chaoyang District was mainly concentrated in the west, while the evaporation from Haidian District was mainly concentrated in the east and south, that from Shijingshan District was concentrated in the south and center, and that from Fengtai District was concentrated in the northeast. When converting the amount of human body evaporation into the unit area, the amount of evaporation in the six core districts of Beijing reached 5075.2 m^3^/km^2^.

## 4. Discussion

**Figure 5:**
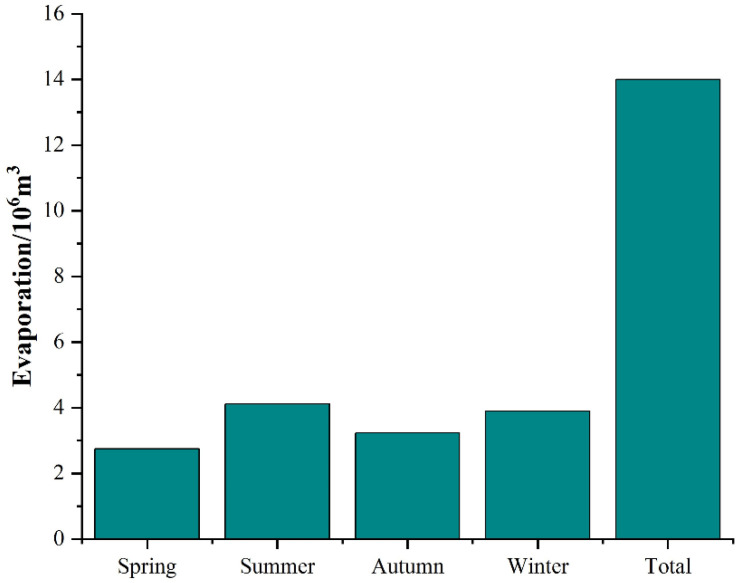
Evaporation from human body in Beijing.

Through accumulating the amount of human body evaporation in each district of Beijing, the amount of human body evaporation in 2020 is shown in Figure 5. The human body evaporation in Beijing reached 2.75, 4.12, 3.23, 3.90 million m^3^ in spring, summer, autumn, and winter, respectively. When integrating the four seasons, the annual figure in Beijing reached 14.0 million m^3^. To further quantify the amount of evaporation generated by the human body in Beijing in 2020, this study compared human body evaporation with the evapotranspiration of trees. The zonal vegetation types in Beijing are warm-temperate deciduous broad-leaved forests, with temperate coniferous forests. According to the data from the census of gardens, broad-leaved trees account for more than 80% of the total number of trees. Acer truncatum is one of the common broad-leaved trees in Beijing, with most trees being between 14–24 cm in diameter [35], so plants with a 15 cm diameter at breast height were chosen as examples, and the annual evapotranspiration per plant is available from relevant research [36]. According to the afforestation technical regulations promulgated by the People’s Republic of China [37], the afforestation density of Acer truncatum for timber was obtained, and its evapotranspiration per unit area was calculated, as shown in Table 2. The annual evapotranspiration of each tree reached 1667.6 kg, and the annual evapotranspiration of the Acer truncatum forest per square kilometer reached 133,408 m^3^. By comparison, the annual human body perspiration in Beijing was 14.0 million m^3^, which was equivalent to the annual evapotranspiration of 104.92 km^2^ of Acer truncatum forest.

The urban water system is a complex system involving the natural water cycle and the influence of artificial factors, and evaporation is a crucial part of it, which is often overlooked [38,39]. In order to further quantify the evapotranspiration produced by the human body, this study compared the calculated evapotranspiration with the urban average annual evaporation. The distribution of the evaporation produced by the human body was uneven. According to our calculation, the higher the population density, the higher the evaporation, and it ranged between 0–78 mm/year in Beijing. Converting the evaporation generated by the human body into the unit area of the urban land, the average was about 10.37 mm/year. Cong et al. [33] used the SEBS model to simulate the evaporation in the urban area of Beijing from 2003–2012, and the annual evaporation was 348 mm/year. Comparing human body evaporation with natural urban evaporation, the evaporation generated by the human body was equivalent to 3% of the natural evapotranspiration in the urban area. Cao et al. [40] performed simulations based on the SEBS model to predict the annual evaporation in Tianjin from 2015 to 2017, and the average annual evaporation value was about 485 mm/year. The evaporation generated by the human body was almost 2.2% of the natural evapotranspiration in Tianjin.

When reflecting on the amount of human body evaporation generated in Beijing in 2020, this study referred to the annual total water use data of some European cities in 2015 and compared it with the amount of human body evaporation generated in Beijing. The relevant data sources are from each country’s official website of the Bureau of Statistics [41,42,43,44], as shown in Table 3. The total water uses of the European cities of Kronoberg, Esbjerg, Aust-Agder, and Tartu were 17.42, 14.30, 15.67, and 6.75 million m^3^, respectively, in 2015. Thus, the 14.0 million m^3^ of water produced through perspiration in Beijing was equivalent to the total annual water uses of Esbjerg and Aust-Agder, and almost twice the annual water use of Tartu.

**Figure 6:**
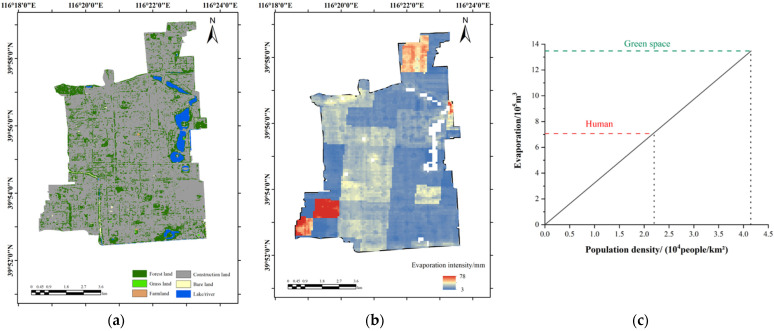
Land use types and evaporation from human body in Xicheng District. (**a**) Land use types; (**b**) evaporation distribution map; (**c**) comparison between human and green space evaporation.

Further, the Xicheng and Shijingshan districts were selected in order to analyze the impact of population density on human body evaporation. According to the land use data, which were obtained from the ESA WorldCover database with a resolution of 10 m × 10 m (https://esa-worldcover.org/en, accessed on 15 January 2022), the land structure was divided to get the green space area in the built-up area. Assuming that the green space was planted with Acer truncatum, based on the evaporation data in Table 1, the annual evaporation from green space was calculated and compared with human body evaporation. Figure 6a–c show the land use types, human body evaporation distribution, and the comparison between the green space and human body evaporation in Xicheng District in 2020, respectively. From Figure 6b, the human body evaporation in Xicheng District was 3–78 mm per square meter, and that in the southwest and north sides were significantly greater than in other areas. The annual human body evaporation in Xicheng District was summarized and compared with the annual evaporation from green space, as shown in Figure 6c. At the current population density of 21,800 people/km^2^ in Xicheng District, this reached 707.3 thousand m^3^. Meanwhile, evaporation from green space reached 1347.8 thousand m^3^, which was equivalent to the amount of human body evaporation in Xicheng District, at a population density of 41,500 people/km^2^.

**Figure 7:**
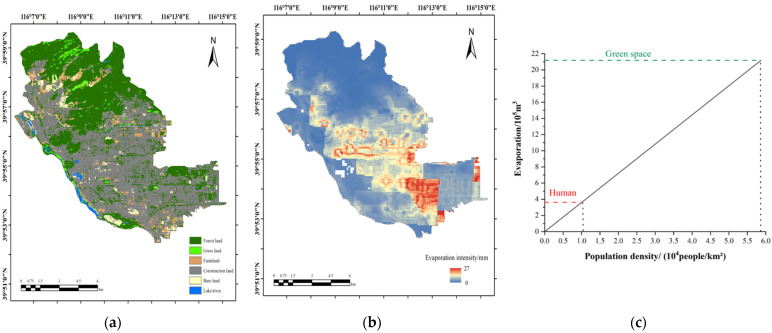
Land use types and evaporation from human body in Shijingshan District. (**a**) Land use types; (**b**) evaporation distribution map; (**c**) comparison between human and green space.

Figure 7a–c show the land use types, human body evaporation distribution, and the comparison between the green space and human body evaporation in Shijingshan District in 2020, respectively. From Figure 7b, we can see that the human body evaporation in Shijingshan District was 0–27 mm per square meter, significantly smaller than in Xicheng District. Similarly, the human body evaporation in Shijingshan District was summarized and compared with the green space evaporation in the built-up area, as shown in Figure 7c. At the current population density of 10,100 people/km^2^ in Shijingshan District, the human body evaporation reached 363.1 thousand m^3^. The evaporation from green space reached 2118.5 thousand m^3^, which was equivalent to the amount of human evaporation in Shijingshan District at a population density of 58,700 people/km^2^. The above comparison illustrates that the amount of evaporation produced by the human body cannot be ignored. With the process of urbanization, the urban population density will continue to increase, and the evaporation produced by the human body in urban areas will gradually approach, or even exceed, the evaporation produced by green space, which will have a significant impact on the water vapor balance in urban areas.

The study calculated the amount of evaporation produced by the human body from the perspective of homogenization based on the hypotheses, and the quantitative results were obtained. Considering the differences between individuals, the results were supplemented and improved. The “China Sleep Research Report 2022” shows that most Chinese people go to bed at 22:00–24:00 p.m. and get up at 6:00–8:00 a.m., with the length of sleeping being between 6–10 h. According to the data released in the “2020 National Fit-ness Activity Survey Bulletin”, it is estimated that the average daily activity time in China is 0.5–2 h, which was the moderate work in this study. Taking the time allocation of each type of activity above, the calculation and analysis of human body evaporation was carried out. Based on the current research result of 14.0 million m^3^, the range of change is from −11.71% to 4.5%.

Human body evaporation is a significant part of indoor water vapor. Zhou et al. [9] compared urban buildings to “trees” cast by reinforced concrete based on the principle of bionics, and the doors and windows of buildings are like the pores of these concrete “trees”, which are the channels for indoor and outdoor water vapor transfer. The indoor and outdoor water vapor transfer is essentially the circulation of air, and the indoor and outdoor temperature gradient is the main driving force. There are many factors that affect the temperature gradient, which can be analyzed from the outside of the building, the inside of the building, and the building itself. For outside the building, solar radiation [45] is an important factor affecting indoor temperature, and the solar irradiance in different regions is affected by the local atmospheric transparency, atmospheric pressure, and geographic location. The outdoor temperature [46] is an important indicator with which to characterize the indoor thermal environment. It mainly transfers heat to the indoor area by thermal convection heat transfer to change the indoor temperature. Building infiltration wind [47] refers to the direct generation of unorganized air flow in the room by building openings or by gaps in the envelope, thereby affecting the indoor temperature. For inside the building, the indoor heat source [48] mainly comes from the heating terminal, indoor lighting, the human body, indoor equipment, etc., and the heat emitted from the indoor heat source affects the indoor temperature. In addition, the material, structure, and geographic location of the building itself also affect the indoor temperature [49,50]. Regarding the indoor and outdoor water vapor transfer efficiency, it may be related to different building types, air circulation, indoor and outdoor relative humidity, etc., and as a next step, this could be explored by monitoring the indoor and outdoor temperatures and by humidity experiments.

In the present study, the weight loss of the human body during sleep was regarded as “insensible perspiration” [51]. Some relevant studies [52,53] have shown that the “insensible perspiration” of the human body is stable between 20 and 50 g/h when the ambient temperature is 20–34 °C. Through comparison, it can be assumed that the results in this paper are consistent with the range of previous studies [52,53]. The calculation results in this paper are also consistent with the result of the previous test study, where the average daily water evaporation per person was 1480 ± 230 mL [54]. Currently, the research on human body perspiration has been expanded to include many fields, such as aerospace, military, clinical medicine, and clothes, but most of the research focuses on evaporation per individual. Based on the definition of the urban water cycle, this study calculated human evaporation on a much greater scale, and the results indicated that the evaporation generated by the human body is considerable. From the calculations, we can see that the amount of human body perspiration in Beijing even exceeded the annual total water use of some less populated European cities, so it is worth considering in the study of evapotranspiration in urban areas in the future, especially in densely populated cities. The mechanism of human body perspiration is relatively complicated, as it is affected by external and internal factors, such as environmental temperature and humidity, age and gender, human body skin temperature and humidity, body metabolic rate, etc. [55]. Therefore, the differences between individuals should be taken into consideration to make the calculation of human body evaporation more accurate in future research.

## 5. Conclusions

In the present study, human body evaporation was defined as the water evaporation induced by human perspiration and respiration. Based on PW sweat prediction equations, the human body evaporation in Beijing was quantified, and several main conclusions were drawn as follows.

(1) Affected by factors such as temperature and the clothing worn, there was a significant seasonal difference in the human body evaporation in Beijing. The highest amount occurred in summer, followed by winter, autumn, and spring.
(2) According to the population distribution, the human body evaporation in six core districts of Beijing was converted into the evaporation intensity, which ranged between 0 to 78 mm/year and decreased from the center to the surroundings. Additionally, the amount of evaporation in the six core districts of Beijing reached 5075.2 m^3^/km^2^.
(3) The total human body evaporation in Beijing was 14.0 million m^3^ in 2020, which was equivalent to the annual evapotranspiration of an Acer truncatum forest area of 104.92 km^2^, comprising trees whose diameters were 15 cm at breast height, with an afforestation density of 800 plants/hm^2^. This exceeded the annual total water use of some European cities, which shows the huge amount of human body evaporation in megacities. From the comparison between the evaporation of human bodies and that of green spaces in the Xicheng and Shijingshan districts, it was found that the human body evaporation in the two districts reached 52.48% and 17.14% of the green space evaporation, respectively, proving that human body evaporation is considerable.
(4) With the progress of urbanization, the population will continue to congregate in urban areas. In addition, the amount of evaporation produced by the human body should be considered when calculating water vapor balance and water flux for densely populated cities. To calculate the evaporation generated by the human body more precisely, the differences between individuals, such as age and other physiological factors, should be taken into consideration in future research.

## Figures and Tables

**Table 1:** Parameters used within the calculation.

**Type of Activity**	**M (W/m^2^)**	**h_c_ (W/(m^2^·K))**	**f_r_**
Sleep	40	2.7	0.35
Light work	75	4.0	0.7
Moderate work	220	8.2	0.73
**Season**	**f_cl_**	**I_cl_ (m^2^·K/W)**	**i_cl_**
Spring	1.22	0.89	0.5
Summer	1.1	0.36	0.55
Autumn	1.28	1.01	0.48
Winter	1.33	1.20	0.43

**Table 2:** Annual evapotranspiration of Acer truncatum.

Diameter at Breast Height (cm)	Afforestation Density (Plants/hm^2^)	Evapotranspiration per Plant (kg/year)	Evapotranspiration (m^3^/km^2^)
15	800	1667.6	133,408

**Table 3:** Total water use of some European cities in 2015.

City	Kronoberg	Esbjerg	Aust-Agder	Tartu
Country	Sweden	Denmark	Norway	Estonia
Total water use (million m^3^)	17.42	14.30	15.67	6.75

## Data Availability

Not applicable.
